# Commentary: Comparison of the Protective Effects of Individual Components of Particulated *trans*-Sialidase (PTCTS), PTC and TS, against High Cholesterol Diet-Induced Atherosclerosis in Rabbits

**DOI:** 10.3389/fcvm.2018.00171

**Published:** 2018-12-03

**Authors:** Maria de Lourdes Higuchi

**Affiliations:** Faculdade de Medicina, Hospital das Clínicas, Instituto do Coraçao, Universidade de São Paulo, São Paulo, Brazil

**Keywords:** microvesicles, atherosclerosis, components of particulated *trans*-sialidase, anti-oxidant natural nanoparticles, PTCTS

In this comment we develop the concept that atherosclerosis is an inflammatory process, aggravated with the presence of infectious agents, such as Coxsackie B virus, HIV, *H.pilori*, CMV, *Mycoplasma pneumoniae*, and *Chlamydophila pneumoniae* and pathogenic archaea. We also develop hypothesis about how PTCTS works, a new natural nutricosmetic produced by nanoparticles derived from orchid flowers, transialidase, and thermal water, which had a strong anti-oxidant effect. In previous work, published *BioMed Research International*, we demonstrated that serum extracellular vesicles containing *Mycoplasma pneumoniae* lipoproteins increased with cholesterol enriched diet, and they decreased with administration of oral PTCTS (experimental work approved by the Ethics Committee of the School of Medicine of University of Sao Paulo). The hypothesis is that, PTCTS would remove pathogenic archaea, the symbiotic microbe responsible for maintenance of other bacteria growing. Then, removing archaea, the macrophages would re-establishes their capacity of killing intra-cellular microbes, decreasing atherosclerotic plaques, in rabbits. Now we intend to initiate human clinical trials to determine safety and efficacy of oral PTCTS in humans.

Aging cells and organisms accumulate increased levels of nuclear DNA damaged by oxidants (1). Numerous pathogens have been identified as contributing factors to the chronic inflammatory state of atherosclerosis such as Coxsackie B virus, HIV, H.pilori, CMV (2, 3). In this general comment we develop the concept about mechanism of PTCTS works, a new anti-oxidant natural nanoparticles (PTCTS) for decreasing the amount of atherosclerotic plaques, possibly due to removal of infectious agents (4).

*Mycoplasma pneumoniae* and *Chlamydophila pneumoniae* (5) were found in human atherosclerotic plaques, and increased levels of anti-*Chlamydophila pneumoniae* and anti-*Mycoplasma.pneumoniae* antibodies were seen after acute myocardial infarction (6, 7). Extracellular vesicles are membrane-coated vesicles, may play a role in endothelial dysfunction, platelet activation and free radical production, classified as microvesicles (0.1 to 1 μm) or exosomes (< 0.1 μm), having procoagulant and proinflammatory properties according to their lipid and protein compositions (8, 9). Microvesicles isolated from human atherosclerotic lesions are highly thrombogenic (10–12). Increased levels of circulating microvesicles in Metabolic Syndrome patients induced *in vitro* endothelial cells studies, NO reduction and superoxide anion production (13), suggesting that microvesicles may be participating on LDL oxidation to generate inflammation and activation of the immune system.

Human coronary arteries with unstable plaques are richer in microvesicles, sometimes containing archaeal DNA, suggesting a possible infectious etiology (14).

Archaea are primitive microorganisms producing collagenase and inflammation, reducing metals such as Fe and Mn (15, 16). Archaeal superoxide dismutases, would neutralize the oxidative capacity of macrophages in the fight against infectious agents (17, 18).

We found greater number of such microvesicles, which had greater quantity of oxLDL and collagenases, on unstable plaque than on normal artery or on stable plaque (19).

PTCTS cosmetic gel was efficient in treatment of lesions by radio dermatitis, with no undesirable side effects (20). The Nutricosmetic with PTCTS, combining transialidase enzyme with nanoparticles derived from orchid extracts, was able to remove archaea. This nutricosmetic agent in experimental work with atherosclerotic rabbits lead to reduction of inflammation in plaques in parallel with removal of microvesicles from serum arteries, without side effects (4, 21).

Nutricosmetics are considered nutritional supplements based on bioactive compounds that have antioxidant, anti-inflammatory, and PTCTS would also remove different infectious agents. In addition to prevent skin aging and strengthening hair and nails, nutricosmetics act in a systemic way, contributing to a better quality of life and well being, under the premise of a healthy body inside and beautiful on the outside. The objective is preventing aging, capillary fall, (22) and atherosclerosis, being necessary the development of human clinical trials to determine efficacy and safety in healthy and atherosclerotic individuals.

## Author contributions

The author confirms being the sole contributor of this work and has approved it for publication.

### Conflict of interest statement

The author declares that the research was conducted in the absence of any commercial or financial relationships that could be construed as a potential conflict of interest.
